# Site of Nerve Division Affects Pain-Related Behavior and Spinal Cord Glial Proliferation after C7 Neurotomy in a Rat Stroke Model

**DOI:** 10.1155/2022/7446482

**Published:** 2022-03-23

**Authors:** Zhenpeng Li, Jintao Fang, Jiantao Yang, Bengang Qin, Wenting He, Jian Qi, Qingtang Zhu, Honggang Wang, Liqiang Gu

**Affiliations:** Department of Microsurgery, Orthopedic Trauma and Hand Surgery, The First Affiliated Hospital, Sun Yat-sen University, 58 Zhongshan 2nd Road, Guangzhou 510080, China

## Abstract

**Objective:**

This study aimed to evaluate whether the site of C7 neurotomy affects spinal cord glial cell activation and pain-related behavior on the paralyzed side in a rat stroke model.

**Methods:**

After middle cerebral artery occlusion (MCAO) was induced in male Sprague-Dawley rats, they underwent C7 neurotomy 0, 2, and 4 mm distal to the intervertebral foramen on the paralyzed side. Pain-related behavior and immunofluorescence examination of spinal cord glial cell activation in the ipsilateral C7 dorsal horn were evaluated.

**Results:**

Mechanical paw withdrawal threshold (MPWT) was lower, and the number of microglia and astrocytes (/mm^2^) was higher as the distance between the site of C7 neurotomy and the intervertebral foramen decreased from 4 mm to 0.

**Conclusion:**

The site of C7 neurotomy affects MPWT and spinal cord glial proliferation in rats with MCAO. Nerve division closer to intervertebral foramen resulted in lower MPWT and higher degree of glial proliferation in the spinal cord.

## 1. Introduction

Stroke is the second leading cause of death worthwhile [1]. Stroke mortality has significantly decreased in conjunction with improvements in treatment. However, many stroke survivors remain permanently disabled [2]. Between 30% and 60% of stroke survivors cannot use their paralyzed hand [3]. A new surgical strategy, contralateral C7 nerve transfer, has been introduced to improve upper limb function in stroke patients with upper limb spastic paralysis. This technique involves transferring the C7 nerve from the contralateral (nonparalyzed) side to the paralyzed side and has achieved considerable improvements in functional outcomes [4–7]. During the operation, the contralateral C7 nerve is sectioned as distally from the spinal cord as possible but proximal to the point at which it combines with other nerves; the ipsilateral C7 nerve (paralyzed side) is sectioned at the intervertebral foramen. Then, the contralateral C7 nerve is drawn through the prespinal route to the paralyzed side and anastomosed directly with the ipsilateral C7 nerve root [8, 9]. However, between 58% and 72% of patients experience neuropathic pain in the paralyzed upper limb after surgery [6, 7]. One cause of this pain may be C7 neurotomy, as many patients experience neuropathic pain after peripheral nerve injury that is characterized by spontaneous pain and mechanical and thermal allodynia. This pain is long lasting, difficult to relieve, and refractory to treatment [10]. After peripheral nerve injury, distal axons on the injured side (distal to the injury) undergo Wallerian degeneration. Both the axon and its myelin sheath are infiltrated with inflammatory cells that release proinflammatory and neural growth factors that augment the perception of painful stimuli [11]. Studies have confirmed that glial cells in the spinal dorsal horn that correspond to the injured nerve, particularly microglia and astrocytes, are rapidly activated [12, 13]. These glial cells play important roles in the initiation and development of neuropathic pain [14]. Specifically, proinflammatory cytokines, chemokines, and neuroactive substances are secreted, which lead to spinal dorsal horn neuron hypersensitivity [15]. A previous rat model study has shown that the site of neurotomy affects corresponding spinal cord motoneuron survival [16]; however, its effect on pain severity has not been examined. This study aimed to explore whether the site of C7 neurotomy affects spinal cord glial cell activation and pain-related behavior on the paralyzed side in rats with middle cerebral artery occlusion (MCAO).

## 2. Materials and Methods

### 2.1. Animals

**Figure 1:**
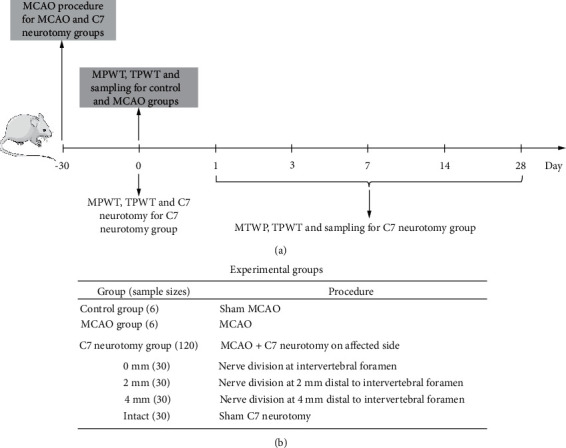
Schematic illustration of the (a) study timeline and (b) experimental groups. MCAO, middle cerebral artery occlusion; MPWT, mechanical paw withdrawal threshold; TPWT, thermal paw withdrawal threshold.

All experiments were approved by the Institutional Animal Care and Use Committee of Sun Yat-Sen University (Approval No. SYSU-IACUC-2021-000205). Male adult Sprague-Dawley rats (specific pathogen free, weight, 280–300 g) were purchased from the Laboratory Animal Center of Sun Yat-Sen University and housed in group of three per cage with comfortable temperature, 12-hour light/dark cycle, and available food and water. Sample size calculations were not performed. Rats that failed stroke modeling were excluded from the study and replaced with condition-matched animals. In total, 170 rats were used, although 38 were excluded because of death (*n* = 33) or mild stroke (*n* = 5). The remaining 132 rats were randomized by drawing lots into three groups: control (*n* = 6), MCAO (*n* = 6), and C7 neurotomy (*n* = 120). The C7 neurotomy group was randomly and equally divided into four subgroups according to site of neurotomy (no division and 0, 2, and 4 mm distal to the intervertebral foramen). A schematic illustration of the study timeline and experimental groups is shown in Figure 1. All operations were performed by an independent operator blinded to the experiment process, and all outcome analyses were performed by independent investigator blinded to the grouping.

### 2.2. MCAO Procedure

MCAO was performed under an operating microscope in the MCAO and C7 neurotomy groups. The procedure has been described in detail by Longa et al. [17]. Briefly, the rats were anesthetized by intraperitoneal injection using 2% pentobarbital sodium (2 ml/kg) and placed in the supine position. Through a midline incision on the anterior neck, the left common carotid artery (CCA), external carotid artery (ECA), and internal carotid artery (ICA) were freed while protecting the vagus nerve. The ECA and its branches were ligated and resected, and the left CCA and ICA were temporarily occluded using microvascular clips. A silicon-coated nylon occlusion suture was inserted into ECA through a small incision in its arterial stump and pushed toward ICA until resistance was encountered. The distance from the tip of the suture to the carotid bifurcation was approximately 18 mm. After 90 min of occlusion, the suture was withdrawn and cerebral blood flow recovered. Sham operation, in which ECA and its branches were only ligated and resected, was performed in the control group. Twenty-four hours after the operation, Longa et al. scale scores [17] were measured in the MCAO and C7 neurotomy groups. Scores from 1 to 3 indicated successful stroke modeling.

### 2.3. C7 Neurotomy Procedure

**Figure 2:**
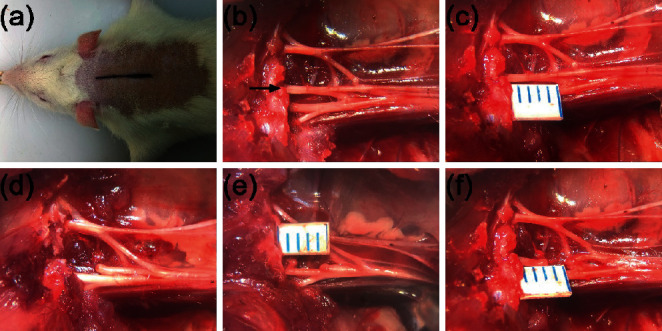
C7 neurotomy procedure. (a) A midline incision on the back of the neck is made. (b) The C5-T1 brachial plexus fully exposed (the black arrow indicates the C7 nerve root). (c) The C7 nerve measured distally relative to the intervertebral foramen (each marking = 1 mm). (d–f) The C7 nerve is resected at 0, 2, or 4 mm according to the subgroup. After neurotomy, the C7 nerve stumps are slightly retracted.

C7 neurotomy was performed under an operating microscope in the C7 neurotomy group rats on day 0. The C7 nerve on the paralyzed side (right) was exposed using the method described by Fang et al. [18]. Briefly, the rats were anesthetized as described above and placed in the prone position. A midline incision on the back of the neck was made to expose the right brachial plexus by separating the superficial and deep neck muscles. After the right scapula was pulled laterally by a retractor, the C7 nerve was carefully identified and freed up to the intervertebral foramen. The C7 nerve was then divided at different sites according to group (no division or 0, 2, or 4 mm distal to the intervertebral foramen). After division, a 2–4 mm distal portion of the nerve was removed to prevent reinnervation. Key steps are shown in Figure 2.

### 2.4. Pain-Related Behavioral Testing

Pain-related behavioral testing included determination of mechanical paw withdrawal threshold (MPWT) and thermal paw withdrawal threshold (TPWT). Rats in all groups were tested on day 0 (day before C7 neurotomy), 1, 3, 7, 14, and 28. Thresholds obtained on day 0 were considered baseline.

### 2.5. MPWT

MPWT of the right forepaw was assessed by the withdrawal response to stimulation by a series of calibrated Von Frey filaments (3.61, 3.84, 4.08, 4.31, 4.56, 4.74, 4.93, and 5.18 mN; Aesthesio, Danmic Global, CA, USA). The 50% withdrawal threshold was determined by the up and down method described by Chaplan et al. [19]. The rats were placed on a metal mesh floor housed in a transparent plastic box. All rats were allowed to adapt to the environment for approximately 15 min before testing. A brisk forepaw withdrawal to the filament was regarded as a positive response. The test was initiated using the filament with 4.31 mN bending force (in the middle of the series); if a withdrawal response was observed, the adjacent weaker filament was used; otherwise, the adjacent stronger filament was used. Lower MPWT indicates greater sensitivity to mechanical stimulation.

### 2.6. TPWT

TPWT of the right forepaw was assessed by measuring the withdrawal response to radiant infrared-heat stimulation using a plantar test apparatus (PL-200, TechMan Co., Chengdu, China). The rats were placed in a glass box and allowed to adapt to the environment for approximately 15 min before testing. The time to withdrawal response after a mobile radiant heat source was positioned under the glass floor and focused on the plantar surface of the forepaw encompassing the glabrous skin was recorded. The time measured before C7 neurotomy was performed and was considered baseline. To avoid potential tissue damage, a 15 s maximal cutoff time was set. Each rat was tested three times at 5 min intervals, and mean time was calculated. Shorter TPWT indicates greater sensitivity to thermal stimulation.

### 2.7. Sampling

All control and MCAO group rats on day 0 and 6 rats in each subgroup of the C7 neurotomy group on days 1, 3, 7, 14, and 28 were randomly selected for brain and spinal cord sampling after the behavioral test. The rats were deeply anesthetized by intraperitoneal injection of 2% pentobarbital sodium (3 ml/kg) before transcardial perfusion of 250 mL of 0.9% saline, followed by 4% paraformaldehyde in 0.1 M phosphate buffer (pH 7.4). The brains were first exposed by craniotomy to confirm focal cerebral infarction, and then, C7 spinal cord segments were harvested by laminectomy. Brain and spinal cord samples were fixed with 4% paraformaldehyde and embedded in paraffin.

### 2.8. Nissl Stain

Brain infarction was evaluated using the Nissl stain. Each brain sample was sliced into 4 *µ*m coronal sections, deparaffinized with xylene, and dehydrated in ethanol. Sections were then incubated with toluidine blue for 2–5 min before differentiation with 0.1% glacial acetic acid. Sections were washed with distilled water until a sharp Nissl body image could be observed under the light microscope and then dehydrated again. Finally, the sections were soaked in xylene for clearance for 10 min and then sealed with neutral resin.

### 2.9. Immunofluorescence

Each C7 spinal cord segment sample was sliced into a series of thirty 4 *µ*m transverse spinal sections. Five equidistant sections were randomly selected. The sections were deparaffinized with xylene and alcohol and immersed in EDTA buffer (pH 8.0) for 15 min at 90°C for antigen retrieval and naturally cooled down to 25°C. After washing for 5 min in 0.01 M phosphate-buffered saline (PBS, pH 7.4) three times, the sections were blocked with 3% bovine serum albumin for 30 min at room temperature and then incubated at 4°C overnight with the following primary antibodies: rabbit antiionized calcium-binding adaptor molecule 1 (Iba-1, for microglia, 1 : 1000, Servicebio Technology Co., Wuhan, China) and rabbit antiglial fibrillary acidic protein (GFAP, for astrocytes, 1 : 1000, Servicebio Technology Co., Wuhan, China). After washing for 5 min in PBS three times, the sections were incubated for 2 h at room temperature with the corresponding secondary antibodies conjugated with Cyrm3 or fluorescein isothiocyanate (1 : 500, Servicebio Technology Co., Wuhan, China). After washing for 5 min in PBS three times, nuclei were counterstained with DAPI (Solarbio Science & Technology Co., Beijing, China) for 15 min. Under a Nikon fluorescence microscope (DS‐U3, Nikon Co., Tokyo, Japan), the microglia and astrocytes were identified by expression of GFAP and Iba-1. For each section, 5 random areas in the dorsal horn of the affected side were examined at ×200 magnification, and the mean number of microglia and astrocytes (expressed as number/mm^2^) in the examined areas was calculated.

### 2.10. Statistical Analysis

Statistical analyses were conducted using SPSS software version 23.0 (IBM Corp., Armonk, NY, USA). Data normality was tested using the Shapiro–Wilk test. The Levene test was used to examine homogeneity of variance. Normally distributed data are expressed as means with standard deviation; skewed data are expressed as medians with interquartile range. The independent *t*-test or Mann–Whitney *U* test was used when performing two-group comparisons as appropriate. For comparison of more than two groups, one-way analysis of variance with the post hoc Tukey test or the Kruskal–Wallis test with post hoc Nemenyi analysis was performed as appropriate. *P* < 0.05 was considered significant.

## 3. Results

### 3.1. General Observations

**Figure 3:**
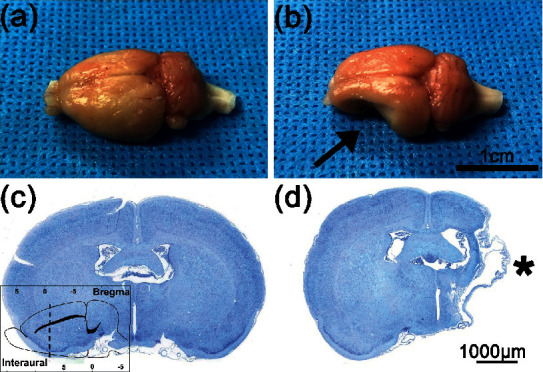
Cortical infarction in rats after the MCAO procedure. (a) Gross brain morphology in the control group. (b) Gross brain morphology in the MCAO and C7 neurotomy groups; the black arrow indicates the area of cortical infarction. Nissl staining in the (c) control group and (d) MCAO and C7 neurotomy groups; the black asterisk indicates the area of cortical infarction (bregma: −0.84 to −1.08 mm; interaural: 8.16–7.92 mm).

**Figure 4:**
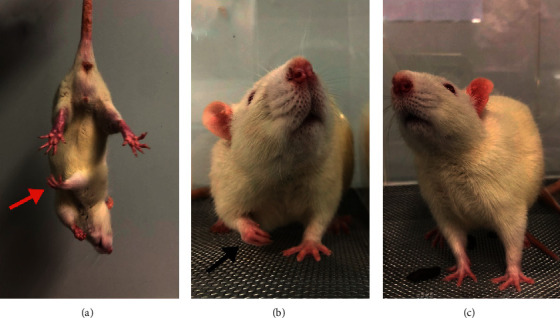
Affected forepaw posture. (a) Thirty days after the MCAO procedure, the rats still could not fully extend the affected forepaw (red arrow). (b) Rats often raised the affected forepaw (black arrow) from the floor and held it when standing or sitting. (c) The abnormal postures caused by C7 neurotomy lasted for approximately one week, but not thereafter.

Following the MCAO procedure, the rats exhibited typical neurologic deficits, such as failure to fully extend the affected forepaw, circling around the paralyzed side, or falling to the paralyzed side. Gross brain morphology and examination of Nissl-stained slides confirmed the existence of focal cerebral infarctions, which were predominantly located in ipsilateral MCA distribution. In the control group, no infarctions were observed (Figure 3). On day 30 after the MCAO procedure, the rats still could not fully extend the affected forepaw (Figure 4(a)).

Following C7 neurotomy, the rats limped and often raised the affected forepaw from the floor and held it when standing or sitting (Figure 4(b)). The abnormal postures caused by C7 neurotomy lasted for only one week approximately (Figure 4(c)). No obvious self-mutilation of the affected limb was observed throughout the experimental period. No abnormality was observed in rats that underwent a sham operation.

### 3.2. Changes in Pain-Related Behavior and Glial Cells after MCAO

**Figure 5:**
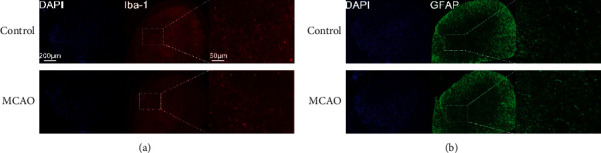
Immunohistochemistry in the C7 dorsal horn of the affected side in the control and MCAO groups. (a) Ionized calcium-binding adaptor molecule 1 (Iba-1) on day 0. (b) Glial fibrillary acidic protein (GFAP) on day 0.

MPWT significantly differed between the control and MCAO groups on day 0 but TPWT did not (Table 1). Mean number of microglia and astrocytes did not significantly differ between groups (Figure 5).

### 3.3. Changes in Pain-Related Behavior and Glial Cells after C7 Neurotomy

Changes in pain-related behavioral testing are given in Tables 2 and 3. Baseline MPWT and TPWT did not significantly differ between subgroups. After C7 neurotomy, MPWT significantly differed between subgroups at each time point, while TPWT did not. MPWT was lower as the distance between the site of C7 neurotomy and the intervertebral foramen decreased from 4 mm to zero. Post hoc MPWT pairwise comparisons between the four subgroups showed a significant difference on days 1 and 3 (*P* < 0.05, Tables S6 and S7); on days 7, 14, and 28, differences were significant for all pairwise comparisons except the following: 0 mm–2 mm, 2 mm–4 mm, and 4 mm–intact (*P* < 0.05, Tables S8–S10).

**Figure 6:**
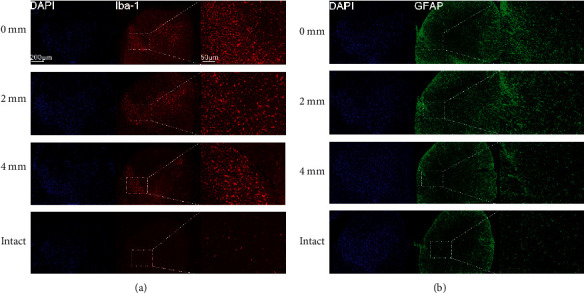
Immunohistochemistry in the C7 dorsal horn of the affected side in the C7 neurotomy subgroups. (a) Ionized calcium-binding adaptor molecule 1 (Iba-1) on day 3. (b) Glial fibrillary acidic protein (GFAP) on day 7.

Changes in mean number of microglia and astrocytes are given in Tables 4 and 5. After C7 neurotomy, the number of microglia increased rapidly. At each time point, mean number significantly differed between the four subgroups, and the number was higher as the distance between the site of C7 neurotomy and the intervertebral foramen decreased from 4 mm to zero. Post hoc pairwise comparisons showed a significant difference between groups on day 1 except for the following: 0 mm–2 mm, 2 mm–4 mm, and 4 mm–intact (*P* < 0.05, Table S11). On the days 3, 7, 14, and 28, the difference for each pairwise comparison was significant (*P* < 0.05, Tables S12–S15). Peak number was observed on day 3, after which the number slowly decreased but remained high until day 28. Astrocyte findings were similar (Tables S16–S20); however, the increase in numbers was slower and the peak was observed on day 7 (Figure 6).

## 4. Discussion

Contralateral C7 nerve transfer has been applied to improve upper limb spastic paralysis after stroke and has achieved good outcomes in terms of releasing spasticity, enhancing muscle strength, and improving voluntary motion coordination [5–7]. The procedure involves sectioning of the C7 nerve at the intervertebral foramen on the paralyzed side, which results in neuropathic pain [6, 7]. We hypothesized that nerve division so close to the spinal cord is the crucial factor. Therefore, we designed an animal experiment to test our hypothesis.

Rats with MCAO induced by the intraluminal filament method have been widely used in animal stroke studies. This model has several advantages: it does not require craniotomy, allows control of embolization duration, and achieves constant cortical infarct area [17, 20]. In humans, ischemic infarctions caused by arterial occlusion account for 87% of strokes [1]. The most common vessel affected is the MCA [21]; therefore, we used a rat MCAO stroke model for our experiments. To avoid the potential impact of maximal spontaneous recovery [22], C7 neurotomy was performed 30 days after MCAO.

Reported incidence rates of central poststroke pain (CPSP), a neuropathic pain syndrome, range between 1% and 14% in stroke survivors [23]. Previous studies have indicated that the development of CPSP in rats is region-specific and associated with a lesion of the ventral posterolateral thalamic nucleus or ventral posteromedial thalamic nucleus [24]. However, secondary thalamic neurodegeneration after MCAO does not induce CPSP-like symptoms in rats [23]. In our study, after the maximal spontaneous recovery process following MCAO, we found a significant difference in MPWT between the control and MCAO groups but not TPWT, indicating that MCAO rats were less sensitive to mechanical stimulation. Therefore, MCAO rats did not develop CPSP-like symptoms. In addition, immunofluorescence showed no difference in mean number of microglia and astrocytes between the groups; therefore, MCAO was not the main factor resulting in the limb pain.

After peripheral nerve injury, Wallerian degeneration occurs in the distal portion of injured axons, which manifests as axonal and myelin sheath degradation and inflammatory cell infiltration. These cells release proinflammatory cytokines, such as interleukins (ILs) and tumor necrosis factor-*α* (TNF-*α*), inflammatory mediators, such as bradykinin and prostaglandins, and nerve growth factor, which lower the pain threshold. In addition, abnormal ectopic excitability causes positive symptoms such as lancinating pain and burning pain [11]. Numerous neuropathic pain models have been previously established using peripheral nerve injury models such as chronic constriction injury [25], partial median and ulnar nerve injury [12], and brachial plexus avulsion injury [13]. These models cause typical symptoms associated with neuropathic pain that has been associated with glial proliferation in the dorsal horn of the spinal cord at the level corresponding to the injured nerve. In our study, the MCAO rats developed symptoms associated with neuropathic pain after C7 neurotomy. Although TPWT changed little, MPWT in the affected forepaw significantly decreased, indicating that the rats were more sensitive to mechanical stimulation. Immunofluorescence showed that microglia and astrocytes in the C7 dorsal horn proliferated rapidly.

Previous studies have shown that microglia and astrocytes in the spinal cord dorsal horn are associated with neuropathic pain. Garrison et al. [26] first reported that astrocyte activation is associated with hyperalgesia. Since then, other studies have confirmed the important role of microglia and astrocytes in the initiation and development of neuropathic pain [12, 13, 27]. In the early stages of neuropathic pain, a variety of neurotransmitter receptors (e.g., glutamate receptors, *γ*-aminobutyric acid receptors, and adrenoreceptors) are rapidly activated by signals released by the affected peripheral nerve endings and neurons in the dorsal horn (e.g., chemokines, neuregulin-1, matrix metalloproteinase-9, and colony stimulating factor-1) [28, 29]. Activated receptors then activate numerous essential intracellular signaling pathways (e.g., the p38 mitogen activated protein kinase, nuclear factor-*κ*B, and phosphoinositide 3-kinase/protein kinase B pathways), which promote proliferation and cause changes in microglial morphology, gene expression, and function of microglia. Microglia release a variety of inflammatory factors (e.g., TNF-*α*, IL-1*β*, IL-6, prostaglandins, and bradykinin), which cause hyperexcitability of neurons in the dorsal horn [30]. Inhibiting the pathways described above has the potential to diminish neuropathic pain [31, 32]. Astrocytes are activated by cytokines released from microglia [33]. In the setting of neuropathic pain, astrocytes also release a variety of inflammatory factors that could be inhibited to decrease neuropathic pain [34]. After the mitogen-activated protein kinase pathway is activated in astrocytes by TNF-*α*, the activated astrocytes secrete monocyte chemoattractant protein-1, which is crucial in the development and maintenance of neuropathic pain [35]. Although activation of astrocytes is later than that of microglia, it lasts for an extended period and plays an important role in the acute-to-chronic pain transition [36]. Therefore, microglia and astrocytes in the dorsal horn might reflect neuropathic pain status. In our study, we found proliferation of microglia and astrocytes in the C7 dorsal horn in MCAO rats after C7 neurotomy, and the microglial proliferation was rapid, which is consistent with previous studies [12, 13]. Moreover, the degree of proliferation was related to the site of nerve division: division closer to the intervertebral foramen resulted in a higher number of microglia and astrocytes in the dorsal horn. Our MPWT experimental results also showed that the closer the division was to the intervertebral foramen, the lower the MPWT was.

Therefore, the site of C7 neurotomy affected pain-related behavior and spinal cord glial proliferation in MCAO rats. These findings may explain the high incidence of neuropathic pain in the paralyzed upper limb in stroke patients who undergo contralateral C7 transfer. Furthermore, they suggest that ipsilateral C7 nerve division should be performed distal to the intervertebral foramen when performing this procedure. However, considering that a long nerve stump may disturb direct anastomosis, determination of the optimal division site requires further investigation.

This study had several limitations. First, sample size calculations and power analysis were not performed. Second, post hoc analysis showed a tendency but no significant difference in the MPWT pairwise comparisons between adjacent C7 neurotomy subgroups (0 mm–2 mm, 2 mm–4 mm, and 4 mm–intact); this may be related to small sample size. The same situation occurred for the microglia and astrocyte analysis. Third, the experiment was based on experimental observations in 28 days after neurotomy; long-term measurements of pain-related behavior and number of glial cells in the spinal cord were not conducted. Fourth, we did not investigate the mechanism underlying our findings.

## 5. Conclusion

The site of C7 neurotomy affects MPWT and spinal cord glial proliferation in rats with MCAO. Nerve division closer to intervertebral foramen resulted in lower MPWT and higher degree of glial proliferation in the spinal cord.

## Figures and Tables

**Table 1:** Changes in pain-related behavioral testing and number of glial cells in the control and middle cerebral artery occlusion groups.

	Control group (95% CI)	MCAO group (95% CI)	*t*/*z*	*P*
MPWT (mN)	3.30 ± 2.42 (0.76–5.83)	25.37 (22.02–25.37) (17.40–28.87)	−2.99 (*z*)	0.030
TPWT (s)	9.53 ± 1.67 (7.78–11.29)	10.13 ± 1.07 (9.01–11.26)	−0.74 (*t*)	0.477
Microglia (amount/mm^2^)	21.50 ± 1.52 (19.91–23.09)	20.33 ± 1.37 (18.90–21.77)	1.40 (*t*)	0.192
Astrocytes (amount/mm^2^)	20.33 ± 1.03 (19.25–21.41)	20.67 ± 1.21 (19.40–21.94)	0.51 (*t*)	0.619

*n* = 6 in each group; *t* value was calculated by the independent *t*-test; *z* value was calculated by the Mann–Whitney *U* test. CI, confidence interval; MPWT, mechanical paw withdrawal threshold; TPWT, thermal paw withdrawal threshold.

**Table 2:** Changes in mechanical paw withdrawal threshold (mN) in the C7 neurotomy subgroups.

Day	0 mm (*n*, 95% CI)	2 mm (*n*, 95% CI)	4 mm (*n*, 95% CI)	Intact (*n*, 95% CI)	*H*	*P*
0	25.37 (25.37–25.37) (30, 21.58–25.34)	25.37 (25.37–25.37) (30, 21.56–25.16)	25.37 (25.37–25.37) (30, 20.91–24.87)	25.37 (25.37–25.37) (30, 22.25–25.19)	0.45	0.929
1	1.90 ± 1.02 (30, 1.52–2.28)	5.45 ± 2.10 (30, 4.67–6.23)	8.69 (6.74–10.36) (30, 7.82–9.29)	25.37 (25.37–25.37) (30, 23.25–25.51)	106.08	<0.001^*∗*^
3	1.52 (1.04–3.33) (24, 1.53–2.48)	5.60 ± 2.03 (24.4.74–6.46)	9.59 ± 2.31 (24, 8.62–10.57)	25.37 (25.37–25.37) (24, 23.84–25.64)	85.27	<0.001^*∗*^
7	1.69 ± 1.03 (18, 1.18–2.20)	5.79 ± 1.87 (18, 4.86–6.72)	10.36 (8.69–15.45) (18, 10.01–13.32)	25.37 (25.37–25.37) (18, 22.67–25.86)	65.17	<0.001^#^
14	1.87 ± 0.99 (12, 1.24–2.50)	6.67 ± 2.28 (12, 5.22–8.12)	9.70 ± 2.63 (12, 8.03–11.37)	25.37 (25.37–25.37) (12, 21.73–26.10)	41.97	<0.001^#^
28	2.24 ± 0.95 (6, 1.24–3.24)	5.68 ± 1.31 (6, 4.30–7.06)	9.39 ± 1.50 (6, 7.82–10.96)	25.37 (23.48–25.37) (6, 20.87–27.34)	21.85	<0.001^#^

Degree of freedom = 3; *H* value was calculated by the Kruskal–Wallis test. ^*∗*^Pairwise post hoc comparisons showed a significant difference (*P* < 0.05) between the subgroups. ^#^Pairwise intergroup differences were significant (*P* < 0.05) except for the following comparisons: 0 mm–2 mm, 2 mm–4 mm, and 4 mm–intact.

**Table 3:** Changes in thermal paw withdrawal threshold (s) in the C7 neurotomy subgroups.

Day	0 mm (*n*, 95% CI)	2 mm (*n*, 95% CI)	4 mm (*n*, 95% CI)	Intact (*n*, 95% CI)	*F*	*P*
0	10.13 ± 1.25 (30, 9.66–10.60)	9.89 ± 1.21 (30, 9.44–10.34)	9.62 ± 0.88 (30, 9.29–9.95)	9.92 ± 1.08 (30, 9.52–10.32)	1.07	0.364
1	9.89 ± 1.46 (30, 9.35–10.43)	9.69 ± 1.11 (30, 9.27–10.10)	9.51 ± 1.02 (30, 9.13–9.89)	9.67 ± 1.06 (30, 9.27–10.07)	0.520	0.670
3	10.19 ± 1.58 (24, 9.52–10.85)	9.94 ± 1.41 (24, 9.34–10.53)	9.51 ± 0.89 (24, 9.14–9.89)	9.81 ± 1.25 (24, 9.27–10.07)	1.11	0.350
7	10.00 ± 1.58 (18, 9.21–10.79)	9.74 ± 1.28 (18, 9.10–10.37)	9.68 ± 1.31 (18, 9.03–10.34)	9.88 ± 1.28 (18, 9.24–10.51)	0.20	0.899
14	9.65 ± 0.70 (12, 9.21–10.09)	9.76 ± 1.27 (12, 8.95–10.57)	9.78 ± 1.06 (12, 9.10–10.45)	9.84 ± 1.38 (12.8.97–10.72)	0.06	0.981
28	9.90 ± 0.54 (6, 9.34–10.46)	9.95 ± 1.32 (6, 8.56–11.33)	9.73 ± 0.59 (6, 9.12–10.35)	10.00 ± 0.94 (6, 9.01–10.99)	0.10	0.960

Degree of freedom = 3; *F* value was calculated by one-way analysis of variance. *P* value of the Levene test: day 0, 0.364; day 1, 0.670; day 3, 0.112; day 7, 0.960; day 14, 0.253; day 28, 0.494.

**Table 4:** Changes in mean number of microglia (/mm^2^) in the C7 neurotomy subgroups.

Day	0 mm (95% CI)	2 mm (95% CI)	4 mm (95% CI)	Intact (95% CI)	*F*/*H*	*P*
1	35.50 (35.00–37.00) (34.80–36.97)	31.83 ± 1.94 (29.80–33.87)	24.17 ± 1.47 (22.62–25.71)	20.50 ± 1.38 (19.10–21.95)	1.07 (*H*)	<0.001^#^
3	44.33 ± 1.21 (43.06–45.60)	34.33 ± 1.03 (33.25–35.42)	28.00 ± 1.41 (26.52–29.48)	20.33 ± 1.63 (18.62–22.05)	343.80 (*F*)	<0.001^*∗*^
7	43.50 ± 1.87 (41.54–45.46)	34.50 ± 1.05 (33.40–35.60)	27.67 ± 1.03 (26.58–28.75)	20.33 ± 1.21 (19.06–21.60)	327.91 (*F*)	<0.001^*∗*^
14	43.33 ± 1.21 (42.06–44.60)	34.17 ± 0.75 (33.38–34.96)	27.50 ± 1.38 (26.05–28.95)	20.33 ± 1.07 (19.25–21.41)	460.36 (*F*)	<0.001^*∗*^
28	38.67 ± 1.37 (37.23–40.10)	29.00 ± 2.68 (26.19–31.82)	24.17 ± 1.72 (22.36–25.97)	21.00 ± 0.89 (20.06–21.94)	111.15 (*F*)	<0.001^*∗*^

*n* = 6 in each group; degree of freedom = 3; *F* value was calculated by one-way analysis of variance; *H* value was calculated by the Kruskal–Wallis test. *P* value of the Levene test: day 3, 0.593; day 7, 0.581; day 14, 0.550; day 28, 0.559. ^*∗*^Pairwise post hoc comparisons showed a significant difference (*P* < 0.05) between the groups. ^#^Pairwise intergroup differences were significant (*P* < 0.05) except for the following: 0 mm–2 mm, 2 mm–4 mm, and 4 mm–intact.

**Table 5:** Changes in mean number of astrocytes (/mm^2^) in the C7 neurotomy subgroups.

Day	0 mm (95% CI)	2 mm (95% CI)	4 mm (95% CI)	Intact (95% CI)	*F*/*H*	*P*
1	34.00 ± 1.41 (32.52–35.48)	27.67 ± 1.37 (26.23–29.10)	24.00 (23.00–24.00) (23.12–24.21)	20.00 ± 1.41 (18.52–21.49)	21.74 (*H*)	<0.001^#^
3	39.00 ± 1.90 (37.01–40.99)	32.00 ± 1.41 (30.52–33.48)	25.67 ± 1.03 (24.58–26.75)	20.17 ± 1.17 (18.94–21.39)	197.14 (*F*)	<0.001^*∗*^
7	43.33 ± 1.75 (41.50–45.17)	34.00 ± 0.89 (33.06–34.94)	27.33 ± 0.82 (26.48–28.19)	20.33 ± 1.86 (18.38–22.29)	288.08 (*F*)	<0.001^*∗*^
14	41.50 ± 2.43 (38.95–44.05)	33.83 ± 0.75 (33.04–34.62)	27.17 ± 1.72 (25.36–28.79)	20.17 ± 1.17 (18.94–21.39)	185.10 (*F*)	<0.001^*∗*^
28	42.33 ± 2.16 (40.07–44.60)	34.33 ± 1.21 (33.06–35.60)	27.67 ± 1.75 (25.83–29.50)	19.67 ± 1.51 (18.09–21.25)	194.73 (*F*)	<0.001^*∗*^

*n* = 6 in each group; degree of freedom = 3; *F* value was calculated by one-way analysis of variance; *H* value was calculated by the Kruskal–Wallis test. *P* value of the Levene test: day 3, 0.133; day 7, 0.173; day 14, 0.321; day 28, 0.613. ^*∗*^Pairwise post hoc comparisons showed a significant difference (*P* < 0.05) between the groups. ^#^Pairwise intergroup differences were significant (*P* < 0.05) except for the following: 0 mm–2 mm, 2 mm–4 mm, and 4 mm–intact.

## Data Availability

The datasets used and/or analyzed during the current study are available from the corresponding author upon request.

## Supplementary Materials

Supplementary Materials The results of the Shapiro–Wilk test are given in Tables S1–S5, and the results of the post hoc test are given in Tables S6–S20.
