# Supplementary material for: Site of Nerve Division Affects Pain-Related Behavior and Spinal Cord Glial Proliferation after C7 Neurotomy in a Rat Stroke Model
Source: Pain Res Manag. 2022 Mar 23;2022:7446482. doi: 10.1155/2022/7446482 (PMC8967577; doi:10.1155/2022/7446482)
Supplement: Supplementary Materials — The results of the Shapiro–Wilk test are given in Tables S1–S5, and the results of the post hoc test are given in Tables S6–S20. [file 7446482.f1.docx]

**Table S1. P values of normality test in control and MCAO groups**

|  | **Control** | **MCAO** |
| --- | --- | --- |
| **MPWT** | **0.503** | **<0.001** |
| **TPWT** | **0.875** | **0.959** |
| **Number of microglia** | **0.389** | **0.554** |
| **Number of astrocytes** | **0.473** | **0.415** |

**Shapiro-Wilk test**

**Table S2. P values of normality test of MPWT in C7 neurotomy groups**

| **Day** | **0mm** | **2mm** | **4mm** | **Intact** |
| --- | --- | --- | --- | --- |
| **0** | **<0.001** | **<0.001** | **<0.001** | **<0.001** |
| **1** | **0.079** | **0.132** | **0.026** | **<0.001** |
| **3** | **0.008** | **0.855** | **0.253** | **<0.001** |
| **7** | **0.135** | **0.463** | **0.017** | **<0.001** |
| **14** | **0.482** | **0.118** | **0.619** | **<0.001** |
| **28** | **0.672** | **0.163** | **0.122** | **<0.001** |

**Shapiro-Wilk test**

**Table S3. P values of normality test of TPWT in C7 neurotomy groups**

| **Day** | **0mm** | **2mm** | **4mm** | **Intact** |
| --- | --- | --- | --- | --- |
| **0** | **0.194** | **0.846** | **0.217** | **0.073** |
| **1** | **0.104** | **0.841** | **0.315** | **0.053** |
| **3** | **0.901** | **0.101** | **0.149** | **0.451** |
| **7** | **0.305** | **0.603** | **0.101** | **0.749** |
| **14** | **0.571** | **0.746** | **0.226** | **0.903** |
| **28** | **0.412** | **0.353** | **0.674** | **0.133** |

**Shapiro-Wilk test**

**Table S4. P values of normality test of number of astrocytes in C7 neurotomy groups**

| **Day** | **0mm** | **2mm** | **4mm** | **Intact** |
| --- | --- | --- | --- | --- |
| **1** | **0.035** | **0.433** | **0.804** | **0.178** |
| **3** | **0.415** | **0.473** | **0.960** | **0.505** |
| **7** | **0.080** | **0.820** | **0.473** | **0.415** |
| **14** | **0.415** | **0.212** | **0.178** | **0.473** |
| **28** | **0.554** | **0.141** | **0.830** | **0.167** |

**Shapiro-Wilk test**

**Table S5. P values of normality test of number of microglia in C7 neurotomy groups**

| **Day** | **0mm** | **2mm** | **4mm** | **Intact** |
| --- | --- | --- | --- | --- |
| **1** | **0.960** | **0.554** | **0.001** | **0.960** |
| **3** | **0.113** | **0.960** | **0.473** | **0.421** |
| **7** | **0.918** | **0.167** | **0.091** | **0.737** |
| **14** | **0.221** | **0.212** | **0.830** | **0.421** |
| **28** | **0.964** | **0.415** | **0.918** | **0.212** |

**Shapiro-Wilk test**

**Table S6. MPWT (Day 1)**

|  | ***P*** |
| --- | --- |
| **0mm-2mm** | **0.003** |
| **0mm-4mm** | **<0.001** |
| **0mm-Intact** | **<0.001** |
| **2mm-4mm** | **0.048** |
| **2mm-Intact** | **<0.001** |
| **4mm-Intact** | **0.001** |

**Nemenyi analysis**

**Table S7. MPWT (Day 3)**

|  | ***P*** |
| --- | --- |
| **0mm-2mm** | **0.024** |
| **0mm-4mm** | **<0.001** |
| **0mm-Intact** | **<0.001** |
| **2mm-4mm** | **0.041** |
| **2mm-Intact** | **<0.001** |
| **4mm-Intact** | **0.007** |

**Nemenyi analysis**

**Table S8. MPWT (Day 7)**

|  | ***P*** |
| --- | --- |
| **0mm-2mm** | **0.073** |
| **0mm-4mm** | **<0.001** |
| **0mm-Intact** | **<0.001** |
| **2mm-4mm** | **0.060** |
| **2mm-Intact** | **<0.001** |
| **4mm-Intact** | **0.062** |

**Nemenyi analysis**

**Table S9. MPWT (Day 14)**

|  | ***P*** |
| --- | --- |
| **0mm-2mm** | **0.081** |
| **0mm-4mm** | **0.001** |
| **0mm-Intact** | **<0.001** |
| **2mm-4mm** | **0.969** |
| **2mm-Intact** | **0.001** |
| **4mm-Intact** | **0.084** |

**Nemenyi analysis**

**Table S10. MPWT (Day 28)**

|  | ***P*** |
| --- | --- |
| **0mm-2mm** | **0.836** |
| **0mm-4mm** | **0.019** |
| **0mm-Intact** | **<0.001** |
| **2mm-4mm** | **0.836** |
| **2mm-Intact** | **0.019** |
| **4mm-Intact** | **0.836** |

**Nemenyi analysis**

**Table S11. Microglia (Day 1)**

|  | ***P*** |
| --- | --- |
| **0mm-2mm** | **1.000** |
| **0mm-4mm** | **0.022** |
| **0mm-Intact** | **<0.001** |
| **2mm-4mm** | **0.715** |
| **2mm-Intact** | **0.017** |
| **4mm-Intact** | **0.907** |

**Nemenyi analysis**

**Table S12. Microglia (Day 3)**

|  | ***P*** |
| --- | --- |
| **0mm-2mm** | **<0.001** |
| **0mm-4mm** | **<0.001** |
| **0mm-Intact** | **<0.001** |
| **2mm-4mm** | **<0.001** |
| **2mm-Intact** | **<0.001** |
| **4mm-Intact** | **<0.001** |

**Tukey test**

**Table S13. Microglia (Day 7)**

|  | ***P*** |
| --- | --- |
| **0mm-2mm** | **<0.001** |
| **0mm-4mm** | **<0.001** |
| **0mm-Intact** | **<0.001** |
| **2mm-4mm** | **<0.001** |
| **2mm-Intact** | **<0.001** |
| **4mm-Intact** | **<0.001** |

**Tukey test**

**Table S14. Microglia (Day 14)**

|  | ***P*** |
| --- | --- |
| **0mm-2mm** | **<0.001** |
| **0mm-4mm** | **<0.001** |
| **0mm-Intact** | **<0.001** |
| **2mm-4mm** | **<0.001** |
| **2mm-Intact** | **<0.001** |
| **4mm-Intact** | **<0.001** |

**Tukey test**

**Table S15. Microglia (Day 28)**

|  | ***P*** |
| --- | --- |
| **0mm-2mm** | **<0.001** |
| **0mm-4mm** | **<0.001** |
| **0mm-Intact** | **<0.001** |
| **2mm-4mm** | **0.001** |
| **2mm-Intact** | **<0.001** |
| **4mm-Intact** | **0.029** |

**Tukey test**

**Table S16. Astrocytes (Day 1)**

|  | ***P*** |
| --- | --- |
| **0mm-2mm** | **0.842** |
| **0mm-4mm** | **0.019** |
| **0mm-Intact** | **<0.001** |
| **2mm-4mm** | **0.842** |
| **2mm-Intact** | **0.019** |
| **4mm-Intact** | **0.842** |

**Nemenyi analysis**

**Table S17. Astrocytes (Day 3)**

|  | ***P*** |
| --- | --- |
| **0mm-2mm** | **<0.001** |
| **0mm-4mm** | **<0.001** |
| **0mm-Intact** | **<0.001** |
| **2mm-4mm** | **<0.001** |
| **2mm-Intact** | **<0.001** |
| **4mm-Intact** | **<0.001** |

**Tukey test**

**Table S18. Astrocytes (Day 7)**

|  | **FDR-adjusted *P*** |
| --- | --- |
| **0mm-2mm** | **<0.001** |
| **0mm-4mm** | **<0.001** |
| **0mm-Intact** | **<0.001** |
| **2mm-4mm** | **<0.001** |
| **2mm-Intact** | **<0.001** |
| **4mm-Intact** | **<0.001** |

**Tukey test**

**Table S19. Astrocytes (Day 14)**

|  | ***P*** |
| --- | --- |
| **0mm-2mm** | **<0.001** |
| **0mm-4mm** | **<0.001** |
| **0mm-Intact** | **<0.001** |
| **2mm-4mm** | **<0.001** |
| **2mm-Intact** | **<0.001** |
| **4mm-Intact** | **<0.001** |

**Tukey test**

**Table S20. Astrocytes (Day 28)**

|  | ***P*** |
| --- | --- |
| **0mm-2mm** | **<0.001** |
| **0mm-4mm** | **<0.001** |
| **0mm-Intact** | **<0.001** |
| **2mm-4mm** | **<0.001** |
| **2mm-Intact** | **<0.001** |
| **4mm-Intact** | **<0.001** |

**Tukey test**
